# Modulation of innate and learned sexual behaviors by the TRP channel Painless expressed in the fruit fly brain: behavioral genetic analysis and its implications

**DOI:** 10.3389/fnbeh.2014.00400

**Published:** 2014-12-02

**Authors:** Shoma Sato, Toshihiro Kitamoto, Takaomi Sakai

**Affiliations:** ^1^Department of Biological Sciences, Tokyo Metropolitan UniversityHachiouji, Tokyo, Japan; ^2^Department of Anesthesia and Pharmacology, University of IowaIowa City, Iowa, USA; ^3^Interdisciplinary Graduate Programs in Genetics and Neuroscience, University of IowaIowa City, Iowa, USA

**Keywords:** TRP channels, Painless, *Drosophila*, courtship, sexual receptivity, sexual orientation, learning and memory

## Abstract

Transient receptor potential (TRP) channels have attracted considerable attention because of their vital roles in primary sensory neurons, mediating responses to a wide variety of external environmental stimuli. However, much less is known about how TRP channels in the brain respond to intrinsic signals and are involved in neurophysiological processes that control complex behaviors. Painless (Pain) is the *Drosophila* TRP channel that was initially identified as a molecular sensor responsible for detecting noxious thermal and mechanical stimuli. Here, we review recent behavioral genetic studies demonstrating that Pain expressed in the brain plays a critical role in both innate and learned aspects of sexual behaviors. Several members of the TRP channel superfamily play evolutionarily conserved roles in sensory neurons as well as in other peripheral tissues. It is thus expected that brain TRP channels in vertebrates and invertebrates would have some common physiological functions. Studies of Pain in the *Drosophila* brain using a unique combination of genetics and physiological techniques should provide valuable insights into the fundamental principles concerning TRP channels expressed in the vertebrate and invertebrate brains.

## Introduction

The transient receptor potential (TRP) channel superfamily is a diverse group of non-selective cation-permeable ion channels (Montell, 2005b; Pedersen et al., 2005). The first member of this superfamily was identified in the fruit fly *Drosophila melanogaster* as the causative gene for a phototransduction mutant in which photoreceptor potentials abnormally displayed a transient, rather than sustained responses during prolonged light stimuli (Minke et al., 1975; Montell and Rubin, 1989; Minke, 2010). TRP channels are well conserved among distantly related species including yeast and invertebrate as well as vertebrate animals. On the basis of the primary amino acid sequence, members of the TRP channel superfamily are classified into seven subfamilies (Montell, 2005b, 2011): TRPC (canonical), TRPV (vanilloid), TRPM (melastatin), TRPA (ankyrin), TRPN (NOMPC-like), TRPP (polycystin), and TRPML (mucolipin). Although TRP channels play important roles in non-excitable cells, it has become apparent that many TRP channels are predominantly expressed in the nervous system. Up until now, TRP channels have been most extensively studied in sensory neurons, where they serve as cellular sensors, responding to a variety of external stimuli such as light, sound, heat, pheromones, and environmental irritants (Voets et al., 2005; Damann et al., 2008). In addition to the prevalent expression in the sensory systems, TRP channels are widely distributed in the central nervous system (CNS) and implicated in the modulation of certain behaviors (Moran et al., 2004). For example, TRPC4 and TRPC5 in the mouse brain contribute to the regulation of innate fear responses (Riccio et al., 2009, 2014), while TRPC6 and TRPV1 are involved in hippocampus-dependent spatial and/or fear memory (Marsch et al., 2007; Li et al., 2008; Zhou et al., 2008). Nonetheless, our understanding concerning the roles and action mechanisms of brain TRP channels is still notably limited. To fill this significant gap in TRP channel research, comprehensive studies using the model organism *Drosophila melanogaster* hold great promise.

Thirteen TRP channel superfamily genes have been identified in *Drosophila*, and sensory functions modulated by the fly TRP channels have been found to be remarkably similar to those modulated by their mammalian counterparts (Montell, 2005a; Fowler and Montell, 2013). Painless (Pain) is one of the *Drosophila* TRP channels, originally identified as a molecular sensor for noxious thermal and mechanical stimuli in larvae (Tracey et al., 2003). Pain also has various sensory functions in adult flies, including thermal nociception, the detection of aversive wasabi stimuli, and gravity sensing (Al-Anzi et al., 2006; Xu et al., 2006; Sun et al., 2009; Ohashi and Sakai, 2014). These characteristics of Pain have been reviewed in a recent article (Fowler and Montell, 2013). Notably, studies indicate that *pain* is expressed in the adult CNS as well and contributes to brain functions controling different aspects of courtship behavior (Table 1). In this current mini-review, we focus on the functions of Pain in the adult brain and discuss the possible mechanisms by which Pain modulates innate and learned courtship behaviors in* Drosophila*.

**Table 1:** **Functions of Pain expressed in *Drosophila* adult brain**.

**Behavior**	**Loss of function phenotype**	**Brain neurons**	**References**
Female sexual behavior	Sexual receptivity enhancement	GABAergic neurons	Sakai et al. (2009)
		Cholinergic neurons	
		IPCs	Sakai et al. (2014)
Male sexual orientation	Homosexual courtship	Olfactory PNs	Wang et al. (2011)
Courtship memory	LTM defect	MB neurons IPCs	Sakai et al. (2013)

*IPCs, insulin-producing cells; PNs, projection neurons; LTM, long-term memory; MBs, mushroom bodies*.

## Pain-expressing neurons in adult brain

**Figure 1:**
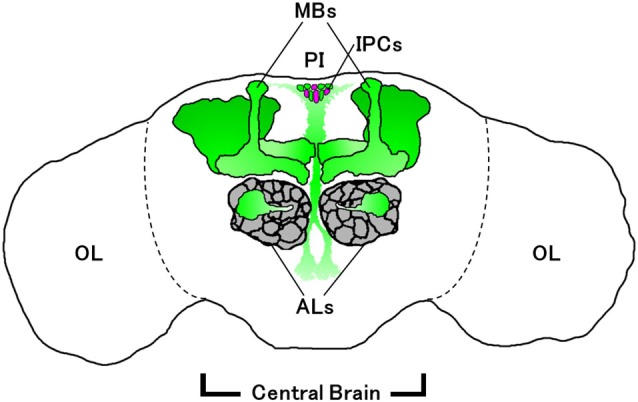
**Schematic diagram of *pain^GAL4^*-expressing neurons (green) in adult brain**. Some of the glomeruli of the antennal lobes (ALs; gray) are required for normal sexual orientation in males (Wang et al., 2011). Mushroom bodies (MBs) and the pars intercerebralis (PI) containing insulin-producing cells (IPCs; magenta) are involved in long-lasting behavioral plasticity (Sakai et al., 2013). IPCs also regulate sexual receptivity in virgin females (Sakai et al., 2014). OL, optic lobe.

Enhancer trapping is often employed in combination with the GAL4/UAS binary gene expression system (Brand and Perrimon, 1993) to visualize *Drosophila* neurons expressing particular genes of interest. A GAL4 enhancer trap line, *pain^GAL4^*, was generated from the *pain^1^* mutant by replacing an EP element in the 5’-flanking region of *pain* with a GAL4-containing P{GawB} transposable element (Tracey et al., 2003). Reporter gene expression driven by *pain^GAL4^* mimics that of the endogenous *pain* mRNA expression in the larval peripheral nervous system (Tracey et al., 2003), suggesting that it is also the case for the adult brain. *pain^GAL4^*-positive neurons are observed in various regions of the adult brain (Al-Anzi et al., 2006; Xu et al., 2006), including mushroom bodies (MBs), which are a brain region important for learning and memory (Figure 1; Heisenberg, 2003; Busto et al., 2010). Similar to larvae, adult flies display characteristic behavioral responses to noxious heat in a Pain-dependent and MB-independent manner (Xu et al., 2006). *pain^GAL4^* also directs reporter gene expression in the olfactory projection neurons (PNs) in antennal lobes (ALs; Wang et al., 2011), the ellipsoid body of the central complex (Sakai et al., 2013, 2014), and the pars intercerebralis (PI) including insulin-producing cells (IPCs; Sakai et al., 2013, 2014).

Several independent analyses further provided evidence of *pain* expression in the adult brain. Microarray and RT-PCR analyses detected endogenous *pain* transcripts in the adult brain extract (Chintapalli et al., 2007; Wang et al., 2011) and *in situ* hybridization analysis revealed a widespread distribution of *pain* transcripts in the neuronal cell body regions of the adult brain (Sakai et al., 2013). Inconsistently, however, anti-Pain immunoreactivity is detected only in the olfactory PNs, not in other brain regions (Wang et al., 2011). The reason for this discrepancy remains unclear. Although *pain* is certainly expressed in the adult brain, further studies are needed to precisely determine brain cells expressing Pain and reveal its intracellular distribution and trafficking.

## Pain regulates sexual behvior in virgin females

Mating behavior in *Drosophila* has been intensively studied since it was first described by Sturtevant (1915). Female-specific sex pheromones elicit courtship behavior from males. Males courting females display a series of stereotypic behavioral elements (Hall, 1994; Emmons and Lipton, 2003). In response to male courtship, females become receptive and accept male courtship. Non-receptive females frequently show various rejection behaviors, such as decamping, wing flicking, kicking or fending off with the legs, curling of the abdomen, and extruding vaginal plates (Spieth, 1952; Ewing, 1983; Hall, 1994). Thus, although males apparently take the initiative in courtship behavior, a female’s decision to accept or reject a male is one of the most important factors for mating success (Ferveur, 2010). Genetic tools available for *Drosophila* studies have been used to elucidate the molecules and neural circuits involved in the regulation of male and female sexual behaviors.

Compared with wild-type virgin females, *pain* mutant females copulate with wild-type males in a shorter time after they are introduced into a small mating chamber (Sakai et al., 2009, 2014). Wild-type males court wild-type and *pain* females to the same extent and there are no particular locomotor defects in *pain* mutant females. Therefore, the rapid copulation of *pain* females is likely caused by their enhanced sexual receptivity, rather than by the improved sex appeal or general inactivity of *pain* females. Experiments using RNA interference (RNAi) with the GAL4/UAS system show that the knockdown of *pain* expression in GABA- or acetylcholine-producing neurons, but not in dopamine- or serotonin-producing neurons, enhances female sexual receptivity as in *pain* mutant females. This result indicates that Pain TRP channels expressed in cholinergic or GABAergic neurons are involved in the regulation of female sexual receptivity (Sakai et al., 2009). In addition, the knockdown of *pain* expression in IPCs, a neuronal subset in the PI, also enhances female sexual receptivity, while the targeted expression of *pain* to IPCs does not restore normal sexual receptivity in *pain* mutant females (Sakai et al., 2014). These results indicate that *pain* expression is required in the GABA- and acetylcholine-producing neurons as well as in IPCs for a normal level of female sexual receptivity. However, *pain* expression in each neuronal subset alone is not sufficient. Interestingly, the disruption of GABAergic transmission or neurosecretion from IPCs has an enhancing effect similar to that of *pain* mutations on female sexual receptivity (Sakai et al., 2009, 2014). Because Pain acts as a Ca^2+^-permeable channel (Sokabe et al., 2008), the suppression of Pain activity likely decreases the intracellular Ca^2+^ level in GABAergic neurons and IPCs. Consequently, the release of GABA and insulin-like peptides from these neurons may be reduced, resulting in the enhanced sexual receptivity observed in *pain*-deficient females. Alternatively, there is the possibility that Pain TRP may function as an anchoring protein and regulate signaling pathways in GABAergic neurons and IPCs. In *Drosophila* photoreceptor cells, TRP plays a role as an anchoring protein, in addition to its function as a Ca^2+^-permeable ion channel. This unconventional function of TRP is required for correct intracellular localization of the scaffold protein, inactivation-no-afterpotential D (INAD; Tsunoda et al., 2001). If Pain also acts as a molecular anchor in GABAergic neurons and IPCs, *pain* mutations or knockdown may induce misregulation of relevant signaling pathways and enhanced sexual receptivity.

## Pain is required for normal sexual orientation in males

Wild-type *Drosophila* males strongly prefer females as their sexual partner and male-male homosexual courtship rarely happens under ordinary conditions (Yamamoto et al., 1996). However, frequent homosexual courtship can be induced by acutely disrupting synaptic transmission from particular neuronal subsets (Kitamoto, 2002), indicating that relevant neuronal circuits are normally involved in suppressing courtship toward other males. Pain TRP channels likely contribute to the activity of such inhibitory circuits because aberrant homosexual courtship is observed more frequently in *pain* mutant males than in wild-type males (Wang et al., 2011). As *pain* mutants show weakened olfactory sensitivity at least to the odorant 4-methyl-cyclohexanol (MCH), the male-male courtship phenotype of *pain* mutants is suggested to be caused by defects in their olfaction (Wang et al., 2011). Consistently, *pain^GAL4^* drives reporter gene expression in the olfactory system including PNs of the ALs (Wang et al., 2011). The specific downregulation of *pain* in PNs leads to the male-male courtship phenotype similar to that of *pain* mutants, while rescue experiments demonstrated that the expression of the wild-type *pain* in the PNs inhibits male-male courtship induced by *pain* mutations. These results suggest that the expression of Pain in the PNs is necessary and sufficient for the suppression of male–male courtship behavior (Wang et al., 2011). In *Drosophila*, a volatile sex pheromone, 11-cis-vaccenyl acetate (cVA), produced by male flies suppresses male-male courtship (Ejima et al., 2007; Ziegler et al., 2013). Thus, it is likely that Pain TRP channels in the PNs are involved in cVA-dependent olfactory processing to prevent homosexual courtship. In addition to the PNs, gustatory receptor neurons (GRNs) likely contribute to homosexual courtship displayed by *pain* mutants because the GRN-specific knockdown of *pain* expression leads to homosexual courtship. However, the GRN-specific knockdown is less effective than the PN-specific knockdown, and the targeted expression of the wild-type *pain* in GRNs does not rescue the male-male courtship phenotype of *pain* mutants. These results suggest that Pain channels expressed in the brain (i.e., PNs), rather than in the sensory neurons (i.e., GRNs), play a primary role in the suppression of male-male courtship.

## Pain regulates long-lasting courtship memory

*Drosophila* male courtship involves a plastic aspect and can be modified by previous sexual experience (Siegel and Hall, 1979; Griffith and Ejima, 2009). More specifically, when a virgin male is paired with a recently mated female, he initially courts her vigorously, but his courtship activity is significantly reduced after repeatedly receiving courtship rejections from the non-virgin partner. After this aversive experience with a female, which releases courtship-inhibiting chemicals, the male fly shows reduced courtship activity even toward a virgin female. The behavioral paradigm inducing this experience-dependent behavioral modulation is called courtship conditioning (Griffith and Ejima, 2009). The suppression of courtship after courtship conditioning is apparently based on memory formation because it is not observed in classical memory mutants (Siegel and Hall, 1979; Gailey et al., 1984). After wild-type males are conditioned (i.e., paired with a recently mated female) for 1 h, the courtship activity of these males toward virgin females remains depressed for at least 8 h, but returns to normal in 24 h (short-lasting memory, SLM; Sakai et al., 2013). In contrast, when the conditioning period is more than 7 h, courtship suppression lasts for at least 5d (long-term memory, LTM; Sakai et al., 2004, 2013; Ishimoto et al., 2009). Thus, the stability of the experience-dependent behavioral modification depends on the length of courtship conditioning.

Males heterozygous and homozygous for *pain* mutations are defective in LTM, but their SLM is apparently unaffected (Sakai et al., 2013). The LTM phenotype in *pain* mutants can be rescued by temporarily expressing wild-type *pain* prior to conditioning. These results indicate that the Pain TRP channel is specifically required for long-term courtship memory, and plays a critical role in the physiological process important for the formation of LTM rather than for LTM storage or retrieval. Furthermore, the targeted knockdown of *pain* expression in either MBs or IPCs resulted in defective courtship LTM, indicating that Pain TRP channels in these neuronal subsets in the adult brain are required for experience-dependent neuronal plasticity that leads to LTM formation.

## Possible role of pain in adult brain

In *Drosophila* photoreceptor cells, several molecules have been identified as possible endogenous activators of the light-dependent TRP channels, TRP and TRPL. They include polyunsaturated fatty acids (PUFAs; Chyb et al., 1999), a combination of phosphatidylinositol 4,5-bisphosphate (PIP_2_) and protons (Huang et al., 2010; Hardie and Franze, 2012), and diacylglycerol (DAG; Delgado et al., 2014). In contrast to TRP and TRPL, nothing is known about whether and how the activity of Pain channels is modulated in the brain by endogenous ligands. These questions are of critical importance to fully understand the mechanisms by which sexual behaviors are regulated through the activation or suppression of Pain TRP channels in the brain. Cell-culture-based studies have revealed that mammalian TRPV1 and TRPA1 channels are also directly activated by PUFAs (Matta et al., 2007; Motter and Ahern, 2012). In the mammalian brain, PUFAs are shown to induce TRPV1-dependent synaptic plasticity (Gibson et al., 2008). In *Drosophila*, the intracellular fatty-acid binding protein, which binds lipids such as PUFAs and acts as transporters, is widely expressed in the adult brain and affects sleep and LTM (Gerstner et al., 2011). It would thus be interesting to examine whether certain lipid molecules including PUFAs could serve as endogenous ligands for Pain channels and whether they are involved in the modulation of Pain activity in the *Drosophila* brain.

Another unanswered question concerning brain Pain channels is how their activation leads to the modification of behaviors. The aforementioned studies have revealed that functional Pain expression in the IPCs is required for the regulation of the normal sexual receptivity of female flies and the experience-dependent suppression of male courtship (Sakai et al., 2013, 2014). These results may suggest that Pain TRP channels control both innate and learned aspects of courtship behavior by modulating the secretion of insulin-like peptides from IPCs. Previous genetic analyses demonstrated that insulin signaling plays important roles in the regulation of various behaviors (Corl et al., 2005; Belgacem and Martin, 2006; Stafford et al., 2012). Thus, it is possible that Pain TRP channels also control these insulin-signaling-dependent behaviors by modulating the secretion of insulin-like peptides from IPCs. In mammals, glucose stimulates insulin secretion from the pancreatic β-cells (Henquin, 2000). Uchida et al. (2011) have presented direct evidence that TRPM2 regulates glucose-stimulated insulin secretion with Ca^2+^ influx (Uchida et al., 2011). Similar to mammalian β-cells, *Drosophila* IPCs also respond to glucose through increased intracellular Ca^2+^ concentrations (Kréneisz et al., 2010). Considering our finding that Pain is required in IPCs for normal sexual behaviors, there is the interesting possibility that Pain in IPCs has comparable functions to mammalian TRPM2 in β-cells and plays a role in the regulation of peptide hormone secretion. This possibility needs to be further investigated in the future study of Pain using molecular, cellular, and physiological approaches.

## Future prospects

Taking advantage of the various genetics and physiological approaches uniquely available in *Drosophila*, future studies of Pain in the *Drosophila* brain are expected to provide valuable insights into the evolutionarily conserved, fundamental principles underlying behavioral regulation by brain TRP channels. For example, in order to examine the possibility that Pain channels in IPCs modulate the secretion of insulin-like peptides, the pH-sensitive GFP variant pHluorin (Miesenböck et al., 1998) can be expressed in IPCs as an insulin-like peptide-pHluorin fusion protein using the GAL4/UAS system. Using this approach, it will be possible to examine the effect of *pain* mutations on the trafficking of peptide-containing dense core vesicles.

We also expect that *Drosophila* will be used to elucidate how TRP channels modulate synaptic plasticity in the brain. Activity-dependent synaptic plasticity in the mammalian brain has been extensively studied to reveal the possible molecular mechanisms underlying learning and memory processes (Bliss and Collingridge, 1993; Kemp and Manahan-Vaughan, 2007; Ho et al., 2011). The repetitive stimulation or paired associative stimulation of target brain neurons can enhance or weaken synaptic efficacy leading to long-term potentiation and long-term depression (LTP and LTD, respectively; Bliss and Lomo, 1973; Levy and Steward, 1983; Dudek and Bear, 1992). Interestingly, TRP channels play an important role in the regulation of LTP and LTD in the mammalian brain. For example, the knockout of the *Trpv1* gene attenuates LTP or LTD in mouse hippocampal neurons (Marsch et al., 2007; Gibson et al., 2008), while TRPV1 triggers LTD in the nucleus accumbens in mice (Grueter et al., 2010) and TRPC is required for the induction of cerebellar LTD (Chae et al., 2012). On the basis of our finding that *pain* mutants have memory defects, it is likely that Pain TRP channels also play a role in activity-dependent synaptic plasticity in the *Drosophila* brain. *Ex vivo* brain culture can be used to examine this hypothesis. Ueno et al. (2013) have reported LTP-like plasticity, known as long-term enhancement (LTE), in synapses between AL and MB. They found that Ca^2+^ responses in MB induced by AL-stimulation are enhanced for at least 2 h after the simultaneous associative stimulation of the AL and ascending fibers of the ventral nerve cord (AFV) in an isolated cultured *Drosophila* brain (Ueno et al., 2013). Because olfactory memory formation and LTE at the AL-MB synapses share common physiological and molecular properties, AL-MB LTE is proposed to be a cellular model of *Drosophila* learning and memory. Although it remains to be determined whether AL-MB synapses show LTD-like synaptic plasticity in *Drosophila*, the Ca^2+^ imaging of an isolated cultured brain will be useful for determining whether Pain TRP channels in AL and MB have an essential role in activity-dependent synaptic plasticity similarly to LTP and LTD in the mammalian brain.

## Conflict of interest statement

The authors declare that the research was conducted in the absence of any commercial or financial relationships that could be construed as a potential conflict of interest.

## References

[B1] Al-Anzi B. Tracey W. D. Jr. Benzer S. (2006). Response of *Drosophila* to wasabi is mediated by *painless*, the fly homolog of mammalian TRPA1/ANKTM1. Curr. Biol. 16, 1034–1040. 10.1016/j.cub.2006.04.002 16647259

[B2] Belgacem Y. H. Martin J. R. (2006). Disruption of insulin pathways alters trehalose level and abolishes sexual dimorphism in locomotor activity in *Drosophila*. J. Neurobiol. 66, 19–32. 10.1002/neu.20193 16187303

[B3] Bliss T. V. Collingridge G. L. (1993). A synaptic model of memory: long-term potentiation in the hippocampus. Nature 361, 31–39. 10.1038/361031a0 8421494

[B4] Bliss T. V. Lomo T. (1973). Long-lasting potentiation of synaptic transmission in the dentate area of the anaesthetized rabbit following stimulation of the perforant path. J. Physiol. 232, 331–356. 4727084 10.1113/jphysiol.1973.sp010273 PMC1350458

[B5] Brand A. H. Perrimon N. (1993). Targeted gene expression as a means of altering cell fates and generating dominant phenotypes. Development 118, 401–415. 8223268 10.1242/dev.118.2.401

[B6] Busto G. U. Cervantes-Sandoval I. Davis R. L. (2010). Olfactory learning in *Drosophila*. Physiology (Bethesda) 25, 338–346. 10.1152/physiol.00026.2010 21186278 PMC3380424

[B7] Chae H. G. Ahn S. J. Hong Y. H. Chang W. S. Kim J. Kim S. J. (2012). Transient receptor potential canonical channels regulate the induction of cerebellar long-term depression. J. Neurosci. 32, 12909–12914. 10.1523/JNEUROSCI.0073-12.2012 22973014 PMC6703793

[B8] Chintapalli V. R. Wang J. Dow J. A. (2007). Using FlyAtlas to identify better *Drosophila melanogaster* models of human disease. Nat. Genet. 39, 715–720. 10.1038/ng2049 17534367

[B9] Chyb S. Raghu P. Hardie R. C. (1999). Polyunsaturated fatty acids activate the *Drosophila* light-sensitive channels TRP and TRPL. Nature 397, 255–259. 10.1038/16703 9930700

[B10] Corl A. B. Rodan A. R. Heberlein U. (2005). Insulin signaling in the nervous system regulates ethanol intoxication in *Drosophila melanogaster*. Nat. Neurosci. 8, 18–19. 10.1038/nn1363 15592467

[B11] Damann N. Voets T. Nilius B. (2008). TRPs in our senses. Curr. Biol. 18, R880–R889. 10.1016/j.cub.2008.07.063 18812089

[B12] Delgado R. Muñoz Y. Peña-Cortés H. Giavalisco P. Bacigalupo J. (2014). Diacylglycerol activates the light-dependent channel TRP in the photosensitive microvilli of *Drosophila melanogaster* photoreceptors. J. Neurosci. 34, 6679–6686. 10.1523/JNEUROSCI.0513-14.2014 24806693 PMC6608135

[B13] Dudek S. M. Bear M. F. (1992). Homosynaptic long-term depression in area CA1 of hippocampus and effects of *N*-methyl-D-aspartate receptor blockade. Proc. Natl. Acad. Sci. U S A 89, 4363–4367. 10.1073/pnas.89.10.4363 1350090 PMC49082

[B14] Ejima A. Smith B. P. Lucas C. van der Goes van Naters W. Miller C. J. Carlson J. R.. (2007). Generalization of courtship learning in *Drosophila* is mediated by *cis*-vaccenyl acetate. Curr. Biol. 17, 599–605. 10.1016/j.cub.2007.01.053 17363250 PMC1913718

[B15] Emmons S. W. Lipton J. (2003). Genetic basis of male sexual behavior. J. Neurobiol. 54, 93–110. 10.1002/neu.10163 12486700

[B16] Ewing A. W. (1983). Functional aspects of *Drosophila* courtship. Biol. Rev. Camb. Philos. Soc. 58, 275–292 10.1111/j.1469-185x.1983.tb00390.x

[B17] Ferveur J. F. (2010). *Drosophila* female courtship and mating behaviors: sensory signals, genes, neural structures and evolution. Curr. Opin. Neurobiol. 20, 764–769. 10.1016/j.conb.2010.09.007 20934322

[B18] Fowler M. A. Montell C. (2013). *Drosophila* TRP channels and animal behavior. Life Sci. 92, 394–403. 10.1016/j.lfs.2012.07.029 22877650 PMC3524398

[B19] Gailey D. A. Jackson F. R. Siegel R. W. (1984). Conditioning mutations in *Drosophila melanogaster* affect an experience-dependent behavioral modification in courting males. Genetics 106, 613–623. 17246201 10.1093/genetics/106.4.613 PMC1202293

[B20] Gerstner J. R. Vanderheyden W. M. Shaw P. J. Landry C. F. Yin J. C. (2011). Fatty-acid binding proteins modulate sleep and enhance long-term memory consolidation in *Drosophila*. PLoS One 6:e15890. 10.1371/journal.pone.0015890 21298037 PMC3029266

[B21] Gibson H. E. Edwards J. G. Page R. S. Van Hook M. J. Kauer J. A. (2008). TRPV1 channels mediate long-term depression at synapses on hippocampal interneurons. Neuron 57, 746–759. 10.1016/j.neuron.2007.12.027 18341994 PMC2698707

[B22] Griffith L. C. Ejima A. (2009). Courtship learning in *Drosophila melanogaster*: diverse plasticity of a reproductive behavior. Learn. Mem. 16, 743–750. 10.1101/lm.956309 19926779 PMC4419844

[B23] Grueter B. A. Brasnjo G. Malenka R. C. (2010). Postsynaptic TRPV1 triggers cell type-specific long-term depression in the nucleus accumbens. Nat. Neurosci. 13, 1519–1525. 10.1038/nn.2685 21076424 PMC3092590

[B24] Hall J. C. (1994). The mating of a fly. Science 264, 1702–1714. 10.1126/science.8209251 8209251

[B25] Hardie R. C. Franze K. (2012). Photomechanical responses in *Drosophila* photoreceptors. Science 338, 260–263. 10.1126/science.1222376 23066080

[B26] Heisenberg M. (2003). Mushroom body memoir: from maps to models. Nat. Rev. Neurosci. 4, 266–275. 10.1038/nrn1074 12671643

[B27] Henquin J. C. (2000). Triggering and amplifying pathways of regulation of insulin secretion by glucose. Diabetes 49, 1751–1760. 10.2337/diabetes.49.11.1751 11078440

[B28] Ho V. M. Lee J. A. Martin K. C. (2011). The cell biology of synaptic plasticity. Science 334, 623–628. 10.1126/science.1209236 22053042 PMC3286636

[B29] Huang J. Liu C. H. Hughes S. A. Postma M. Schwiening C. J. Hardie R. C. (2010). Activation of TRP channels by protons and phosphoinositide depletion in *Drosophila* photoreceptors. Curr. Biol. 20, 189–197. 10.1016/j.cub.2009.12.019 20116246

[B30] Ishimoto H. Sakai T. Kitamoto T. (2009). Ecdysone signaling regulates the formation of long-term courtship memory in adult *Drosophila melanogaster*. Proc. Natl. Acad. Sci. U S A 106, 6381–6386. 10.1073/pnas.0810213106 19342482 PMC2669368

[B31] Kemp A. Manahan-Vaughan D. (2007). Hippocampal long-term depression: master or minion in declarative memory processes? Trends Neurosci. 30, 111–118. 10.1016/j.tins.2007.01.002 17234277

[B32] Kitamoto T. (2002). Conditional disruption of synaptic transmission induces male-male courtship behavior in *Drosophila*. Proc. Natl. Acad. Sci. U S A 99, 13232–13237. 10.1073/pnas.202489099 12239352 PMC130616

[B33] Kréneisz O. Chen X. Fridell Y. W. Mulkey D. K. (2010). Glucose increases activity and Ca^2+^ in insulin-producing cells of adult *Drosophila*. Neuroreport 21, 1116–1120. 10.1097/WNR.0b013e3283409200 20890228 PMC3075590

[B34] Levy W. B. Steward O. (1983). Temporal contiguity requirements for long-term associative potentiation/depression in the hippocampus. Neuroscience 8, 791–797. 10.1016/0306-4522(83)90010-6 6306504

[B35] Li H. B. Mao R. R. Zhang J. C. Yang Y. Cao J. Xu L. (2008). Antistress effect of TRPV1 channel on synaptic plasticity and spatial memory. Biol. Psychiatry 64, 286–292. 10.1016/j.biopsych.2008.02.020 18405883

[B36] Marsch R. Foeller E. Rammes G. Bunck M. Kössl M. Holsboer F.. (2007). Reduced anxiety, conditioned fear and hippocampal long-term potentiation in transient receptor potential vanilloid type 1 receptor-deficient mice. J. Neurosci. 27, 832–839. 10.1523/jneurosci.3303-06.2007 17251423 PMC6672910

[B37] Matta J. A. Miyares R. L. Ahern G. P. (2007). TRPV1 is a novel target for omega-3 polyunsaturated fatty acids. J. Physiol. 578, 397–411. 10.1113/jphysiol.2006.121988 17038422 PMC2075157

[B38] Miesenböck G. De Angelis D. A. Rothman J. E. (1998). Visualizing secretion and synaptic transmission with pH-sensitive green fluorescent proteins. Nature 394, 192–195. 10.1038/28190 9671304

[B39] Minke B. (2010). The history of the *Drosophila* TRP channel: the birth of a new channel superfamily. J. Neurogenet. 24, 216–233. 10.3109/01677063.2010.514369 21067449 PMC3103766

[B40] Minke B. Wu C. Pak W. L. (1975). Induction of photoreceptor voltage noise in the dark in *Drosophila* mutant. Nature 258, 84–87. 10.1038/258084a0 810728

[B41] Montell C. (2005a). *Drosophila* TRP channels. Pflugers Arch. 451, 19–28. 10.1007/s00424-005-1426-2 15952038

[B42] Montell C. (2005b). The TRP superfamily of cation channels. Sci. STKE 2005:re3. 10.1126/stke.2722005re3 15728426

[B43] Montell C. (2011). The history of TRP channels, a commentary and reflection. Pflugers Arch. 461, 499–506. 10.1007/s00424-010-0920-3 21287198

[B44] Montell C. Rubin G. M. (1989). Molecular characterization of the *Drosophila trp* locus: a putative integral membrane protein required for phototransduction. Neuron 2, 1313–1323. 10.1016/0896-6273(89)90069-x 2516726

[B45] Moran M. M. Xu H. Clapham D. E. (2004). TRP ion channels in the nervous system. Curr. Opin. Neurobiol. 14, 362–369. 10.1016/s0959-4388(04)00070-4 15194117

[B46] Motter A. L. Ahern G. P. (2012). TRPA1 is a polyunsaturated fatty acid sensor in mammals. PLoS One 7:e38439. 10.1371/journal.pone.0038439 22723860 PMC3378573

[B47] Ohashi H. Sakai T. (2014). Novel behavioral assay of wasabi avoidance in *Drosophila melanogaster* (Diptera: Drosophilidae) using a video tracking system. Appl. Entomol. Zool. 10.1007/s13355-014-0302-y

[B48] Pedersen S. F. Owsianik G. Nilius B. (2005). TRP channels: an overview. Cell Calcium 38, 233–252. 10.1016/j.ceca.2005.06.028 16098585

[B49] Riccio A. Li Y. Moon J. Kim K. S. Smith K. S. Rudolph U.. (2009). Essential role for TRPC5 in amygdala function and fear-related behavior. Cell 137, 761–772. 10.1016/j.cell.2009.03.039 19450521 PMC2719954

[B50] Riccio A. Li Y. Tsvetkov E. Gapon S. Yao G. L. Smith K. S.. (2014). Decreased anxiety-like behavior and Galphaq/11-dependent responses in the amygdala of mice lacking TRPC4 channels. J. Neurosci. 34, 3653–3667. 10.1523/JNEUROSCI.2274-13.2014 24599464 PMC3942581

[B51] Sakai T. Kasuya J. Kitamoto T. Aigaki T. (2009). The *Drosophila* TRPA channel, painless, regulates sexual receptivity in virgin females. Genes Brain Behav. 8, 546–557. 10.1111/j.1601-183X.2009.00503.x 19531155 PMC2728068

[B52] Sakai T. Sato S. Ishimoto H. Kitamoto T. (2013). Significance of the centrally expressed TRP channel *painless* in *Drosophila* courtship memory. Learn. Mem. 20, 34–40. 10.1101/lm.029041.112 23247253 PMC3533128

[B53] Sakai T. Tamura T. Kitamoto T. Kidokoro Y. (2004). A clock gene, *period*, plays a key role in long-term memory formation in *Drosophila*. Proc. Natl. Acad. Sci. U S A 101, 16058–16063. 10.1002/0471684228.egp02386 15522971 PMC528738

[B54] Sakai T. Watanabe K. Ohashi H. Sato S. Inami S. Shimada N.. (2014). Insulin-producing cells regulate the sexual receptivity through the painless TRP channel in *Drosophila* virgin females. PLoS One 9:e88175. 10.1371/journal.pone.0088175 24505416 PMC3913769

[B55] Siegel R. W. Hall J. C. (1979). Conditioned responses in courtship behavior of normal and mutant *Drosophila*. Proc. Natl. Acad. Sci. U S A 76, 3430–3434. 10.1073/pnas.76.7.3430 16592682 PMC383839

[B56] Sokabe T. Tsujiuchi S. Kadowaki T. Tominaga M. (2008). *Drosophila* painless is a Ca^2+^-requiring channel activated by noxious heat. J. Neurosci. 28, 9929–9938. 10.1523/JNEUROSCI.2757-08.2008 18829951 PMC6671277

[B57] Spieth H. T. (1952). Mating behavior within the genus *Drosophila* (Diptera). Bull. Am. Mus. Nat. Hist. 99, 395–474.

[B58] Stafford J. W. Lynd K. M. Jung A. Y. Gordon M. D. (2012). Integration of taste and calorie sensing in *Drosophila*. J. Neurosci. 32, 14767–14774. 10.1523/JNEUROSCI.1887-12.2012 23077061 PMC6621435

[B59] Sturtevant A. H. (1915). A sex-linked character in *Drosophila* repleta. Am. Nat. 49, 189–192 10.1086/279474

[B60] Sun Y. Liu L. Ben-Shahar Y. Jacobs J. S. Eberl D. F. Welsh M. J. (2009). TRPA channels distinguish gravity sensing from hearing in Johnston’s organ. Proc. Natl. Acad. Sci. U S A 106, 13606–13611. 10.1073/pnas.0906377106 19666538 PMC2717111

[B61] Tracey W. D. Jr. Wilson R. I. Laurent G. Benzer S. (2003). *painless*, a *Drosophila* gene essential for nociception. Cell 113, 261–273. 10.1016/S0092-8674(03)00272-1 12705873

[B62] Tsunoda S. Sun Y. Suzuki E. Zuker C. (2001). Independent anchoring and assembly mechanisms of INAD signaling complexes in *Drosophila* photoreceptors. J. Neurosci. 21, 150–158. 11150331 10.1523/JNEUROSCI.21-01-00150.2001 PMC6762460

[B63] Uchida K. Dezaki K. Damdindorj B. Inada H. Shiuchi T. Mori Y.. (2011). Lack of TRPM2 impaired insulin secretion and glucose metabolisms in mice. Diabetes 60, 119–126. 10.2337/db10-0276 20921208 PMC3012163

[B64] Ueno K. Naganos S. Hirano Y. Horiuchi J. Saitoe M. (2013). Long-term enhancement of synaptic transmission between antennal lobe and mushroom body in cultured *Drosophila* brain. J. Physiol. 591, 287–302. 10.1113/jphysiol.2012.242909 23027817 PMC3630786

[B65] Voets T. Talavera K. Owsianik G. Nilius B. (2005). Sensing with TRP channels. Nat. Chem. Biol. 1, 85–92. 10.1038/nchembio0705-85 16408004

[B66] Wang K. Guo Y. Wang F. Wang Z. (2011). *Drosophila TRPA* channel *painless* inhibits male-male courtship behavior through modulating olfactory sensation. PLoS One 6:e25890. 10.1371/journal.pone.0025890 22073144 PMC3206795

[B67] Xu S. Y. Cang C. L. Liu X. F. Peng Y. Q. Ye Y. Z. Zhao Z. Q.. (2006). Thermal nociception in adult *Drosophila*: behavioral characterization and the role of the *painless* gene. Genes Brain Behav. 5, 602–613. 10.1111/j.1601-183x.2006.00213.x 17081265

[B68] Yamamoto D. Ito H. Fujitani K. (1996). Genetic dissection of sexual orientation: behavioral, cellular and molecular approaches in *Drosophila melanogaster*. Neurosci. Res. 26, 95–107. 10.1016/s0168-0102(96)01087-5 8953572

[B69] Zhou J. Du W. Zhou K. Tai Y. Yao H. Jia Y.. (2008). Critical role of TRPC6 channels in the formation of excitatory synapses. Nat. Neurosci. 11, 741–743. 10.1038/nn.2127 18516035

[B70] Ziegler A. B. Berthelot-Grosjean M. Grosjean Y. (2013). The smell of love in *Drosophila*. Front. Physiol. 4:72. 10.3389/fphys.2013.00072 23576993 PMC3617446

